# Nucleolar activity in neurodegenerative diseases: a missing piece of the puzzle?

**DOI:** 10.1007/s00109-012-0981-1

**Published:** 2012-11-20

**Authors:** Rosanna Parlato, Grzegorz Kreiner

**Affiliations:** 1 Department of Molecular Biology of the Cell I, DKFZ-ZMBH Alliance, German Cancer Research Center, Im Neuenheimer Feld, 581, 69120 Heidelberg, Germany; 2 Department of Brain Biochemistry, Institute of Pharmacology, Polish Academy of Sciences, Smetna 12, 31-343, Cracow, Poland

**Keywords:** rRNA, Nucleolus, Cellular stress, Neurodegeneration, Mouse models

## Abstract

Nucleoli are the sites where synthesis of rRNA and ribosomal assembly take place. Along with these “traditional” roles, the nucleolus controls cellular physiology and homeostasis. The cellular and molecular alterations associated with impaired nucleolar activity (“nucleolar stress”) have just started to be systematically explored in the nervous system taking advantage of newly available animal models lacking rRNA synthesis in specific neurons. These studies showed that nucleolar function is necessary for neuronal survival and that its modality of action differs between and within cell types. Nucleolar function is also crucial in pathology as it controls mitochondrial activity and critical stress signaling pathways mimicking hallmarks of human neurodegenerative diseases. This mini-review will focus on the modes of action of nucleolar stress and discuss how the manipulation of nucleolar activity might underscore novel strategies to extend neuronal function and survival.

## Non-traditional roles of the nucleolus under physiological conditions

Nucleoli are non-membrane-bound structures within the nucleus where ribosomal RNA (rRNA) genes are transcribed. These atypical cellular organelles consist of three major compartments with specific functions: (1) fibrillar centers (FC, pre-rRNA synthesis), (2) dense fibrillar components (DFC, pre-RNA processing), and (3) granular components (GC, ribosome assembly). Their organization allows hosting a number of proteins and RNAs with an important role in various cellular processes. Thus the perturbation of nucleolar dynamic assembly exerts profound effects on several cellular functions [1]. A detailed description of nucleolar morphology and organization has been provided elsewhere [1, 2]. Here we focus on the mechanisms by which nucleolar activity may regulate neuronal function and survival and contribute especially to neurodegenerative diseases.

One intriguing mechanism to control embryonic development, differentiation, and survival is the nucleolar shuttling of cell cycle regulators and transcription factors dictating cell lineage to the nucleoplasm [1]. Moreover, rRNA repression seems required for the execution of differentiation programs. For example, during the embryonic development, transcription of rRNA genes is repressed by lineage-commitment transcription factors turning off the pluripotency genes [3]. During neural lineage commitment there is no direct evidence that rRNA transcription is inhibited; it is likely, however, that mechanisms involving nucleolar dynamics could be in play. For example in brain and retina the levels of the nucleolar protein nucleostemin, a controller of pre-rRNA processing, are rapidly reduced before cell cycle exit and neural differentiation [4, 5], suggesting that rRNA biosynthesis may have a regulatory effect on neurogenesis.

However, the major regulatory function associated with altered dynamics of nucleolar proteins is connected to the stress response and involves among others the release of ribosomal proteins (RPs) to the nucleoplasm. In response to adverse growth conditions, metabolic deficits, and oxidative stress, rRNA synthesis is downregulated by mechanisms involving transcription factors and epigenetic modifications [6]. This perturbation of nucleolar activity and integrity has been defined “nucleolar stress” [7]. Disruption of ribosome biogenesis releases RPs such as L5, L11, L23, and S7 into the nucleoplasm where they interfere with the activity of the E3 ubiquitin ligase Mdm2. Normally, this enzyme promotes proteasomal degradation of the transcription factor p53, but this function is impaired by RPs leading to accumulation of p53, which in turn initiates transcriptional and non-transcriptional programs [8]. By sensing cellular stress signals and transmitting them to the p53 stabilization system the nucleolus plays a fundamental role as a “stress sensor” [9]. Nucleolar stress may affect p53 levels also by non-RPs. The nucleolar protein nucleophosmin is dowregulated upon excitotoxic stimuli in neurons and alters p53 levels. Nevertheless, nucleophosmin-induced cell death appears to be p53 independent [10], suggesting that other yet unknown pathways control the stress response.

More regulatory functions can be postulated based on the observation that the nucleoli are also composed of RNAs, which are involved in the processing and maturation of cellular RNAs. For example, small nuclear RNAs are modified in the nucleolus, suggesting a possible link between nucleolar activity and splicing regulation [11]. In mature neurons changes in protein synthesis during synaptic activity are linked to increased number of nucleoli, by the postsynaptic protein AIDA-1d that regulates the generation of functional nucleoli by enhancing the release of small nuclear ribonucleoproteins (snRNPs) to the nucleoli [12].

More recently, a significant number of small non-coding RNAs (ncRNAs) regulating mRNA translation have been located in the GC of the nucleolus [13]. Although the role of this nucleolar transit is not understood, it is tempting to speculate that the regulation of microRNAs (miRNAs) processing and location is an additional mechanism linking nucleolar activity to protein synthesis regulation. Recently, it has been demonstrated that also specific stress stimuli such as acidosis and heat shock [14] induce immobilization of proteins by long ncRNAs in the nucleolus. In turn, reduced levels of ribosomal proteins may alleviate miRNA-mediated repression of translation initiation [15], highlighting the finely tuned cross-talk of the translational components that have their epicenter in the nucleolus. These premises and the increasing evidence of the role of ncRNAs in neuronal development and plasticity [16] support the unexplored functional link between nucleolus and ncRNAs in neuronal survival and activity.

In summary, by sequestering regulatory proteins and RNAs, and by influencing their dynamics upon specific stimuli, the nucleolus can finely tune distinct cellular functions beyond protein synthesis under physiological conditions.

## Disruption of nucleolar activity in neurodegenerative diseases

It is well known that nucleolar malfunction contributes to the pathology of several rare human genetic disorders, such as Werner syndrome, dyskeratosis congenita, Treacher Collins syndrome and predisposes to certain forms of cancer [17, 18]. Moreover, decline in rRNA synthesis and nucleolar size occur during aging, which is the principal risk factor for neurodegenerative diseases [19, 20]. One of the major problems in neurodegenerative diseases is that the majority of cases are sporadic and, even when an inherited basis is ascertained, there is a high inter-individual variability. Thus, major efforts are directed to identify factors influencing disease onset and progression. Current treatments only help to ameliorate the symptoms but treatments that stop or reverse the pathology are still missing.

**Fig. 1:**
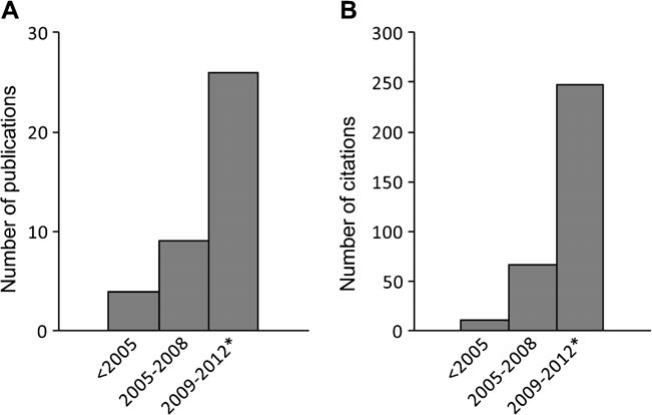
Diagram showing the increasing number of publications and citations related to nucleolar stress. **a** Number of publications found in PubMed database searching for “nucleolar + stress”. Only four out of 39 publications have been published before the year 2005. **b** Number of times the publications in (**a**) have been cited; asterisk, last updated on Aug 31, 2012

The interest on nucleolar stress is gaining momentum and the number of studies is rapidly growing over the last years [21] (Fig. 1). A focus on the role of nucleolar stress for neuronal activity and survival offers a basic and yet transformative perspective to the field of research on neurodegeneration. For example, silencing of rDNA may occur during early stages of Alzheimer´s disease (AD) pathology and play a role in AD-related ribosomal deficits and, ultimately, in dementia [22]. In line with this speculation, differential methylation activity of the human rDNA has been proposed as a mechanism to decrease rRNA gene expression in AD patients. Thus, rDNA specific methylation pattern could be used as a marker of the disease or of its progression [22].

Nucleolar integrity is disrupted in dopaminergic neurons of Parkinson´s disease (PD) patients in human post-mortem brain samples [23]. Treatment of mice with the neurotoxin MPTP (widely used in pharmacological models of PD to inhibit complex I activity in dopaminergic neurons), decreases rRNA synthesis causing early nucleolar disruption [23]. The findings demonstrating that impaired mitochondrial activity affects nucleolar function support the concept that nucleolar stress may occur at early disease stages and that contributes to the pathogenesis. Although the molecular basis of this control is still not understood, regulators of rRNA transcription might be involved. In fact, previous studies identified nucleolin as a protein-regulating rRNA synthesis and ribosome biogenesis [24, 25] and interacting with alpha-synuclein and DJ-1, two major proteins involved in familial PD pathogenesis. Strikingly, the expression levels of nucleolin are dramatically reduced in the substantia nigra pars compacta of human PD patients [26].

More recently, misfolded DJ-1 protein caused by L166P mutation has been shown to alter rRNA biogenesis in cellular models of PD [27], further supporting the association of impaired nucleolar activity with PD pathogenesis.

Nucleolar activity and structure are altered also in trinucleotide repeat disorders. These neurodegenerative diseases are associated with expansion of trinucleotide in repeats transcribed or untranscribed region of different genes. Huntington’s disease (HD), for instances, is associated with an expansion in exon 1 of the huntingtin gene, which leads to an aberrant polyglutamine tract in the huntingtin protein. Several studies have shown that transcription of rRNA genes is altered in HD. Rrs1 (regulator of ribosome synthesis), a protein inhibiting transcription of both rRNA and ribosomal protein genes, is highly expressed in a presymptomatic HD mouse model [28]. The levels of the basal RNA polymerase I factor UBF1 are decreased in cellular and animal HD models [29]. In post-mortem specimens of human HD cases, insoluble aggregates of huntingtin—a hallmark of this disorder—are found in the nucleolus [30]. The presence of insoluble protein aggregates is not limited to HD and is a rather common feature of neurodegenerative diseases. For instances, aggregates are found in the nucleolus of spinocerebellar ataxia patients, an inherited disorder caused by CAG or CTG expansion [30, 31]. In these neurodegenerative diseases nucleolin appears to play a role because CAG RNAs deprive rRNA promoter of this histone chaperone, downregulating rRNA transcription [32]. Abnormal interaction with nucleolin also accounts for the neurological disorder ataxia with oculomotor apraxia type 1, due to mutated aprataxin [33]. In several human cancers the angiogenic ribonuclease angiogenin (ANG) is upregulated and acts as a transcription factor, binding to rRNA promoters and stimulating rRNA transcription. A deficiency in ANG instead causes amyotrophic lateral sclerosis and degeneration of motor neurons [34]. Moreover, nucleolar activity may be repressed by inhibition of the proteasomal machinery, another hallmark of neurodegenerative diseases. RNA protein aggregates form within the nucleolus and are dependent on nucleolar integrity [35].

Dysfunction of snRNP biogenesis involves protein relocalization to the nucleolus in type I spinal muscular atrophy, an autosomal recessive disorder leading to degeneration and death of motor neurons caused by loss or mutations of the survival motor neuron 1 gene [36].

Mutant RNA molecules downregulating rRNA transcription in polyglutamine diseases [32] and mutant proteins altering the localization of nucleolar proteins in PD [27] show an active role of mutant gene products to cause rRNA transcription failure, supporting similar mechanistic studies in other neurodegenerative disorders. The association of nucleolar stress with neurodegenerative diseases raises the question of what cellular and molecular alterations depend on it.

## A lesson from mouse models with nucleolar stress in specific populations of neurons: similarities with neuropathological conditions

**Fig. 2:**
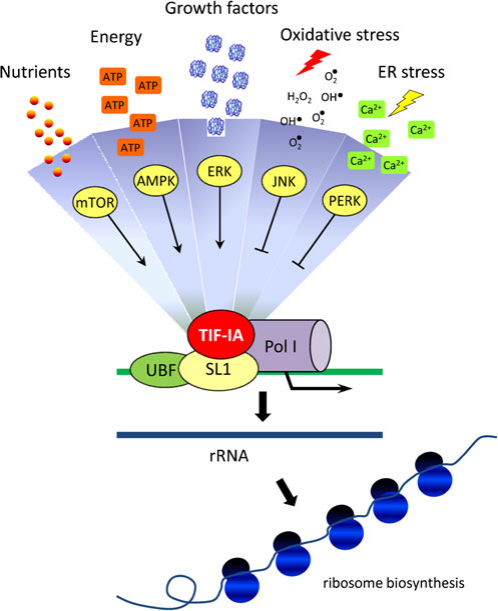
Schematic of the RNA polymerase I machinery at the rDNA promoters with the different signals and pathways regulating TIF-IA activity and exerting positive or negative control on nucleolar activity and integrity

Recently developed mouse models in which nucleolar function is specifically impaired have provided the first mechanistic insights into nucleolar stress in the nervous system. These models are based on the genetic ablation of TIF-IA, an evolutionary conserved transcription factor essential for the recruitment of RNA polymerase I to rRNA promoters. Since its identification, it was evident that TIF-IA activity is strongly dependent on external signals [37]. By now, it is known that TIF-IA is regulated by a variety of protein kinases at distinct serine residues: ERK and RSK in response to mitogenic signals [38], S6K in response to growth stimuli [39, 40], JNK2 in response to oxidative stress [41], AMPK in response to cellular energy status [42], PERK-dependent phosphorylation in response to endoplasmic reticulum stress [43] (Fig. 2). In addition, proteasome activity is required for pre-rRNA synthesis and TIF-IA may represent a potential link by which proteasomes are recruited to rRNA genes [44].

TIF-IA global knock-out in mice results in early embryonic lethality [45]. Specific loss of TIF-IA in neural progenitors leads to rRNA synthesis inhibition, nucleolar disruption, rapid apoptotic death of developing neurons, and consequent anencephalia [46]. The conditional ablation of TIF-IA in mice offers the major advantage of allowing focused investigation on the impact of nucleolar stress not only on cell cycle and growth regulation of neural progenitors, but also on quiescent postmitotic neurons in living organisms. Inactivation of the TIF-IA gene in adult dopaminergic neurons by a drug-inducible approach leads to a phenotype closely resembling PD, characterized by depletion of dopamine in the striatum, oxidative stress, mitochondrial impairment, progressive and differential loss of dopaminergic neurons in the substantia nigra pars compacta, as well as marked deficiencies in motor performance [23]. At the mechanistic level, mTOR activity is downregulated by TIF-IA ablation in dopaminergic neurons, underscoring a negative feedback that co-regulate and coordinate the translational process. In addition, the p53-dependent apoptosis in proliferating cells appears to be conserved in dopaminergic neurons under nucleolar stress, as shown by increased neuronal survival in absence of p53 [23].

The importance of p53 for the pathogenesis of neurodegenerative diseases has been clearly shown for HD, and further confirmed also for AD and PD. It is suggested that p53 could also serve as a convergence point in the molecular pathways for different neurodegenerative diseases [47]. p53 induces cell cycle arrest, senescence, and apoptosis in response to different stress signals such as DNA damage, hypoxia, nutrient deprivation, and nucleolar stress [8]. However, identifying the key elements that define a particular p53-mediated stress response outcome remains a central, yet unresolved, question. The identification of such downstream effectors is crucial to manipulate the deleterious consequences of cellular stress mediated by nucleolar disruption.

## Future directions and perspectives

**Fig. 3:**
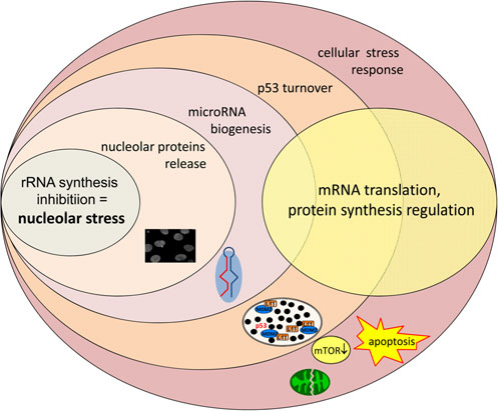
Summary of the cellular and molecular alterations derived from nucleolar stress. The “non-traditional” nucleolar roles are depicted as concentric circles with the early events closer to the nucleolus and intersecting with the “traditional” nucleolar role in protein synthesis

Nucleolar stress may start a progressive series of events affecting protein translation, which can be deregulated in consequence of altered distribution of nucleolar proteins and/or biogenesis of miRNAs, as well as of other cellular mechanisms. Because ribosomes seem to be “specialized” for the synthesis of specific mRNA [48], nucleolar stress by regulating ribosomal composition could specifically affect the translation of particular sets of mRNA in developing and adult neurons. The activation of p53 and the negative feedback on mTOR call in play metabolic and survival pathways executing cell fate decision, by inhibiting mitochondrial function and protein synthesis (Fig. 3).

The “TIF-IA-based models” will allow exploring initial adaptive mechanisms to stress conditions and by indirectly interfering with p53 turnover in specific cells can be used to gain understanding of context-dependent p53 functions. Some attempts have been reported to overexpress p53 in a constitutive and/or inducible way by the generation of transgenic mice [49], but engineered mice having high p53 activity in specific cell types are not available because of the difficulty to generate mutant mice which reproduce the natural levels of proteins. Ultimately, TIF-IA mutant mice can be used to develop and validate therapeutic intervention and identify biomarkers associated with a specific degenerative pattern (Table 1). Modulation of the Pol I transcriptional machinery is already proposed for anticancer therapies either by targeting specific components or the upstream pathways. Inhibition of Pol I in cancer cells has been shown to induce their apoptotic death [50]. Intriguingly, the TIF-IA mutant mice have been already useful for testing the neuroprotective role of the phosphatase PTEN in dopaminergic neurons, supporting their possible use for evaluating new therapeutic approaches [51].

**Table 1:** Potential applications of the different types of TIF-IA mutant mice for the investigation of features linked to nucleolar stress

Type of mutation	Phenotype	Application
Global knock out [45]	Embryonic lethality (E8.5); growth deficits	Study of early embryonic compensatory mechanisms
Embryonic neural progenitors [46]	Anencephalia at birth; impaired neurogenesis (E13.5)	Study of effects on early neuronal differentiation
Adult hippocampal neurons [46]	Progressive but differential degeneration of adult hippocampal neurons	Analysis of changes of neuronal activity and protein synthesis before neuronal death
Embryonic or adult dopaminergic neurons [23, 51]	Parkinsonism; early mitochondrial dysfunction and mTOR downregulation	Search for biomarkers; neurorestorative/neuroprotective strategies
Other neurons	Unpublished observations	Analysis of context-specific responses to nucleolar stress
Overexpression; heterozygosity	not determined yet	Role of nucleolar stress as a disease modifier

Increasing evidence point to a multifactorial basis of neurodegenerative disease onset and progression. Perhaps there is time to bridge the gap between the reductionist approach focused on particular molecules involved in the pathology of a particular disease. The research for identifying common molecular checkpoints as possible new targets for pharmacotherapy seems necessary. Despite the total market of 2.2 billion USD estimated for anti-PD drugs major pharmaceutical companies discouraged by the relatively little progresses announced cuts to brain disorder research in favor of sequencing projects to identify genetic risk factors [52].

Investing more efforts in defining the toxic species causing rRNA transcription failure and the molecular alterations of nucleolar stress in specific cellular contexts will shed new light onto the mechanisms underlying differential vulnerability to stress conditions and indicate potential targets to manipulate them. Otherwise there will always be a missing piece in our understanding of neurological disorders and in our way to cure them.

## References

[1] Boisvert FM, van Koningsbruggen S, Navascues J, Lamond AI (2007). The multifunctional nucleolus. Nat Rev Mol Cell Biol.

[2] Boulon S, Westman BJ, Hutten S, Boisvert FM, Lamond AI (2010). The nucleolus under stress. Mol Cell.

[3] Ali SA, Dobson JR, Lian JB, Stein JL, van Wijnen AJ, Zaidi SK, Stein GS (2012) A Runx2-HDAC1 co-repressor complex regulates rRNA gene expression by modulating UBF acetylation. J Cell Sci 125:2732–2739 10.1242/jcs.100909 PMC3403236 22393235

[4] Paridaen JT, Janson E, Utami KH, Pereboom TC, Essers PB, van Rooijen C, Zivkovic D, MacInnes AW (2011). The nucleolar GTP-binding proteins Gnl2 and nucleostemin are required for retinal neurogenesis in developing zebrafish. Dev Biol.

[5] Lo D, Lu H (2010). Nucleostemin: another nucleolar "Twister" of the p53-MDM2 loop. Cell Cycle.

[6] Grummt I, Ladurner AG (2008). A metabolic throttle regulates the epigenetic state of rDNA. Cell.

[7] Pestov DG, Strezoska Z, Lau LF (2001). Evidence of p53-dependent cross-talk between ribosome biogenesis and the cell cycle: effects of nucleolar protein Bop1 on G(1)/S transition. Mol Cell Biol.

[8] Vousden KH, Lane DP (2007). p53 in health and disease. Nat Rev Mol Cell Biol.

[9] Olson MO (2004). Sensing cellular stress: another new function for the nucleolus?. Sci STKE.

[10] Marquez-Lona EM, Tan Z, Schreiber SS (2012). Nucleolar stress characterized by downregulation of nucleophosmin: a novel cause of neuronal degeneration. Biochem Biophys Res Commun.

[11] Gerbi SA, Borovjagin AV, Lange TS (2003). The nucleolus: a site of ribonucleoprotein maturation. Curr Opin Cell Biol.

[12] Jordan BA, Fernholz BD, Khatri L, Ziff EB (2007). Activity-dependent AIDA-1 nuclear signaling regulates nucleolar numbers and protein synthesis in neurons. Nat Neurosci.

[13] Politz JC, Hogan EM, Pederson T (2009). MicroRNAs with a nucleolar location. RNA.

[14] Audas TE, Jacob MD, Lee S (2012). Immobilization of proteins in the nucleolus by ribosomal intergenic spacer noncoding RNA. Mol Cell.

[15] Janas MM, Wang E, Love T, Harris AS, Stevenson K, Semmelmann K, Shaffer JM, Chen PH, Doench JG, Yerramilli SV (2012). Reduced expression of ribosomal proteins relieves microRNA-mediated repression. Mol Cell.

[16] Bian S, Sun T (2011). Functions of noncoding RNAs in neural development and neurological diseases. Mol Neurobiol.

[17] Johnson FB, Marciniak RA, Guarente L (1998). Telomeres, the nucleolus and aging. Curr Opin Cell Biol.

[18] Montanaro L, Trere D, Derenzini M (2008). Nucleolus, ribosomes, and cancer. Am J Pathol.

[19] Garcia Moreno LM, Cimadevilla JM, Gonzalez Pardo H, Zahonero MC, Arias JL (1997). NOR activity in hippocampal areas during the postnatal development and ageing. Mech Ageing Dev.

[20] Mattson MP, Magnus T (2006). Ageing and neuronal vulnerability. Nat Rev Neurosci.

[21] Hetman M, Pietrzak M (2012). Emerging roles of the neuronal nucleolus. Trends Neurosci.

[22] Pietrzak M, Rempala G, Nelson PT, Zheng JJ, Hetman M (2011). Epigenetic silencing of nucleolar rRNA genes in Alzheimer's disease. PLoS One.

[23] Rieker C, Engblom D, Kreiner G, Domanskyi A, Schober A, Stotz S, Neumann M, Yuan X, Grummt I, Schutz G (2011). Nucleolar disruption in dopaminergic neurons leads to oxidative damage and parkinsonism through repression of mammalian target of rapamycin signaling. J Neurosci.

[24] Cong R, Das S, Ugrinova I, Kumar S, Mongelard F, Wong J, Bouvet P (2012). Interaction of nucleolin with ribosomal RNA genes and its role in RNA polymerase I transcription. Nucleic Acids Res.

[25] Abdelmohsen K, Gorospe M (2012) RNA-binding protein nucleolin in disease. RNA Biol 9:799–808 10.4161/rna.19718 PMC3495746 22617883

[26] Caudle WM, Kitsou E, Li J, Bradner J, Zhang J (2009). A role for a novel protein, nucleolin, in Parkinson's disease. Neurosci Lett.

[27] Vilotti S, Codrich M, Dal Ferro M, Pinto M, Ferrer I, Collavin L, Gustincich S, Zucchelli S (2012). Parkinson's disease DJ-1 L166P alters rRNA biogenesis by exclusion of TTRAP from the nucleolus and sequestration into cytoplasmic aggregates via TRAF6. PLoS One.

[28] Carnemolla A, Fossale E, Agostoni E, Michelazzi S, Calligaris R, De Maso L, Del Sal G, MacDonald ME, Persichetti F (2009). Rrs1 is involved in endoplasmic reticulum stress response in Huntington disease. J Biol Chem.

[29] Lee J, Hwang YJ, Boo JH, Han D, Kwon OK, Todorova K, Kowall NW, Kim Y, Ryu H (2011). Dysregulation of upstream binding factor-1 acetylation at K352 is linked to impaired ribosomal DNA transcription in Huntington's disease. Cell Death Differ.

[30] Latonen L (2011). Nucleolar aggresomes as counterparts of cytoplasmic aggresomes in proteotoxic stress. Proteasome inhibitors induce nuclear ribonucleoprotein inclusions that accumulate several key factors of neurodegenerative diseases and cancer. Bioessays.

[31] Baltanas FC, Casafont I, Weruaga E, Alonso JR, Berciano MT, Lafarga M (2011). Nucleolar disruption and cajal body disassembly are nuclear hallmarks of DNA damage-induced neurodegeneration in purkinje cells. Brain Pathol.

[32] Tsoi H, Lau TC, Tsang SY, Lau KF, Chan HY (2012). CAG expansion induces nucleolar stress in polyglutamine diseases. Proc Natl Acad Sci U S A.

[33] Becherel OJ, Gueven N, Birrell GW, Schreiber V, Suraweera A, Jakob B, Taucher-Scholz G, Lavin MF (2006). Nucleolar localization of aprataxin is dependent on interaction with nucleolin and on active ribosomal DNA transcription. Hum Mol Genet.

[34] Li S, Hu GF (2010). Angiogenin-mediated rRNA transcription in cancer and neurodegeneration. Int J Biochem Mol Biol.

[35] Latonen L, Moore HM, Bai B, Jaamaa S, Laiho M (2011). Proteasome inhibitors induce nucleolar aggregation of proteasome target proteins and polyadenylated RNA by altering ubiquitin availability. Oncogene.

[36] Tapia O, Bengoechea R, Palanca A, Arteaga R, Val-Bernal JF, Tizzano EF, Berciano MT, Lafarga M (2012) Reorganization of Cajal bodies and nucleolar targeting of coilin in motor neurons of type I spinal muscular atrophy. Histochem Cell Biol 137:657–667 10.1007/s00418-012-0921-8 22302308

[37] Bodem J, Dobreva G, Hoffmann-Rohrer U, Iben S, Zentgraf H, Delius H, Vingron M, Grummt I (2000). TIF-IA, the factor mediating growth-dependent control of ribosomal RNA synthesis, is the mammalian homolog of yeast Rrn3p. EMBO Rep.

[38] Zhao J, Yuan X, Frodin M, Grummt I (2003). ERK-dependent phosphorylation of the transcription initiation factor TIF-IA is required for RNA polymerase I transcription and cell growth. Mol Cell.

[39] Mayer C, Zhao J, Yuan X, Grummt I (2004). mTOR-dependent activation of the transcription factor TIF-IA links rRNA synthesis to nutrient availability. Genes Dev.

[40] Grewal SS, Evans JR, Edgar BA (2007). *Drosophila* TIF-IA is required for ribosome synthesis and cell growth and is regulated by the TOR pathway. J Cell Biol.

[41] Mayer C, Bierhoff H, Grummt I (2005). The nucleolus as a stress sensor: JNK2 inactivates the transcription factor TIF-IA and down-regulates rRNA synthesis. Genes Dev.

[42] Hoppe S, Bierhoff H, Cado I, Weber A, Tiebe M, Grummt I, Voit R (2009). AMP-activated protein kinase adapts rRNA synthesis to cellular energy supply. Proc Natl Acad Sci U S A.

[43] DuRose JB, Scheuner D, Kaufman RJ, Rothblum LI, Niwa M (2009). Phosphorylation of eukaryotic translation initiation factor 2alpha coordinates rRNA transcription and translation inhibition during endoplasmic reticulum stress. Mol Cell Biol.

[44] Fatyol K, Grummt I (2008). Proteasomal ATPases are associated with rDNA: the ubiquitin proteasome system plays a direct role in RNA polymerase I transcription. Biochim Biophys Acta.

[45] Yuan X, Zhou Y, Casanova E, Chai M, Kiss E, Grone HJ, Schutz G, Grummt I (2005). Genetic inactivation of the transcription factor TIF-IA leads to nucleolar disruption, cell cycle arrest, and p53-mediated apoptosis. Mol Cell.

[46] Parlato R, Kreiner G, Erdmann G, Rieker C, Stotz S, Savenkova E, Berger S, Grummt I, Schutz G (2008). Activation of an endogenous suicide response after perturbation of rRNA synthesis leads to neurodegeneration in mice. J Neurosci.

[47] Chang JR, Ghafouri M, Mukerjee R, Bagashev A, Chabrashvili T, Sawaya BE (2012). Role of p53 in neurodegenerative diseases. Neurodegener Dis.

[48] Xue S, Barna M (2012). Specialized ribosomes: a new frontier in gene regulation and organismal biology. Nat Rev Mol Cell Biol.

[49] Christophorou MA, Martin-Zanca D, Soucek L, Lawlor ER, Brown-Swigart L, Verschuren EW, Evan GI (2005). Temporal dissection of p53 function in vitro and in vivo. Nat Genet.

[50] Bywater MJ, Poortinga G, Sanij E, Hein N, Peck A, Cullinane C, Wall M, Cluse L, Drygin D, Anderes K (2012). Inhibition of RNA polymerase I as a therapeutic strategy to promote cancer-specific activation of p53. Cancer Cell.

[51] Domanskyi A, Geissler C, Vinnikov IA, Alter H, Schober A, Vogt MA, Gass P, Parlato R, Schutz G (2011). Pten ablation in adult dopaminergic neurons is neuroprotective in Parkinson's disease models. FASEB J.

[52] Abbott A (2011). Novartis to shut brain research facility. Nature.

